# Identification of Gene Expression Biomarkers Predictive of Latent Tuberculosis Infection Using Machine Learning Approaches

**DOI:** 10.3390/genes16060715

**Published:** 2025-06-18

**Authors:** Youssra Boumait, Boutaina Ettetuani, Manal Chrairi, Afaf Lamzouri, Rajaa Chahboune

**Affiliations:** 1 Biology Molecular Unit, Life and Health Sciences Laboratory, Faculty of Medicine and Pharmacy, Abdelmalek Essaâdi University, Tangier 93000, Morocco; chrairi.manal@gmail.com (M.C.); chahboune82rajaa@yahoo.fr (R.C.); 2 Systems and Data Engineering Team, National School of Applied Sciences, Abdelmalek Essaâdi University, Tangier 93000, Morocco; ettetuani.boutaina@gmail.com; 3 Genetic Unit, Life and Health Sciences Laboratory, Faculty of Medicine and Pharmacy, Abdelmalek Essaâdi University, Tangier 93000, Morocco; lamzouriafaf@hotmail.com; 4 Department of Medical Genetics and Oncogenetics, Mohammed VI University Hospital, Tangier 90000, Morocco

**Keywords:** bioinformatics, biomarkers, gene expression, latent tuberculosis infection, machine learning, transcriptomic

## Abstract

Latent tuberculosis infection (LTBi) affects nearly a quarter of the global population, yet current diagnostic methods are limited by low sensitivity and specificity. This study applied an integrative bioinformatics framework, incorporating machine learning techniques, to identify robust gene expression biomarkers associated with LTBi. We analyzed four publicly available transcriptomic datasets from peripheral blood mononuclear cells (PBMCs), representing latent, active, and healthy states. Differentially expressed genes (DEGs) were identified, followed by gene ontology (GO) enrichment, functional clustering, and miRNA interaction analysis. Semantic similarity, unsupervised clustering, and pathway enrichment were applied to refine the gene list. Key biomarkers were prioritized using receiver operating characteristic (ROC) curve analysis, with CCL2 and CXCL10 emerging as top candidates (AUC > 0.85). This multi-step approach demonstrates the potential of combining transcriptomic profiling with established machine learning and bioinformatics tools to uncover candidate biomarkers for improved LTBi detection, and it also provides a foundation for future experimental validation.

## 1. Introduction

Tuberculosis (TB) remains a global health concern, and it is responsible for millions of cases and deaths annually. The disease is caused by Mycobacterium tuberculosis (*M. tuberculosis*), an airborne pathogen that mainly affects the lungs but can spread to other organs [1]. *M. Tuberculosis* is highly adaptable and capable of evading the immune system, allowing it to persist in the host [2]. Following initial infection, the pathogen can either cause active tuberculosis, characterized by symptoms such as persistent cough, fever, and weight loss, or it can enter a latent phase. This latent form of infection, referred to as latent tuberculosis infection (LTBi) [3,4], is defined by the presence of *M. tuberculosis* in the body but without the causing of clinical symptoms or being transmissible to others [5].

Tuberculosis (TB), caused by *Mycobacterium tuberculosis* (Mtb), remains one of the leading infectious causes of death worldwide, with an estimated 10.6 million new cases and 1.3 million deaths reported in 2022 [6]. While active TB attracts clinical attention, latent tuberculosis infection (LTBi) affects nearly one-quarter of the global population and poses a significant public health challenge due to its 5–10% lifetime risk of progression to active disease, especially in immunocompromised individuals [7,8].

LTBi affects approximately one-quarter of the world’s population and poses a significant public health challenge because individuals with LTBi have a 5–10% lifetime risk of developing active TB, especially if their immune system becomes compromised [9]. Identifying biomarkers that can distinguish LTBi from active tuberculosis and predict the risk of progression is critical for targeted treatment strategies and for preventing the spread of TB [10,11].

Despite their widespread use, current diagnostic tools, such as the tuberculin skin test (TST) and interferon-gamma release assays (IGRAs), lack sufficient sensitivity and specificity to distinguish LTBi from active TB [12,13]. This diagnostic gap highlights the need for molecular biomarkers capable of reliably identifying infection states, especially in high-risk populations.

Recent studies have leveraged transcriptomic profiling of peripheral blood mononuclear cells (PBMCs) to identify immune-related gene signatures indicative of TB infection and progression [14,15]. For instance, a 16-gene signature reported by Zak et al. [16] predicted TB progression up to 18 months prior to clinical manifestation. Similarly, Berry et al. [17] demonstrated distinct transcriptional profiles that separate active TB from other inflammatory conditions. However, robust biomarkers specific to LTBi remain insufficiently explored.

Machine learning (ML) techniques, particularly those integrating feature selection and functional enrichment, are powerful tools to identify meaningful patterns within high-dimensional transcriptomic data [18,19]. Unsupervised clustering methods have shown success in stratifying TB patients and discovering novel gene networks involved in host–pathogen interactions [11].

This study addresses the challenge of identifying potential biomarkers for LTBi by analyzing transcriptomic data to uncover differentially expressed genes (DEGs) that may serve as indicators of infection status or progression risk. The analysis focused on gene expression profiles that differentiate LTBi from other stages of TB, such as pulmonary, extrapulmonary, and active infections. By leveraging machine learning techniques for clustering and enrichment analyses, we aim to identify candidate genes that may provide new insights into the biology of LTBi and improve diagnostic accuracy. Then, the aim is to control the spread of *M. tuberculosis*. Many bioinformatics workflows and supporting tools were needed to predict the development of active TB from latent TB infection (LTBi). To compile the studies listed in Appendix A (Table A1), we employed a systematic search strategy across various academic databases, including PubMed, Scopus, and Google Scholar, using keywords such as “latent tuberculosis”, “biomarkers”, “gene expression”, “transcriptomics”, and “tuberculosis diagnosis” [20,21,22]. We established specific inclusion criteria, focusing on studies investigating biomarkers associated with latent tuberculosis infection (LTBi) or those distinguishing between LTBi and active tuberculosis. Eligible studies were required to be original research articles, reviews, or meta-analyses that provide insights into gene expression profiles or other molecular markers relevant to LTBi. We limited our search to studies published within the last decade to ensure the findings were current and pertinent to contemporary tuberculosis research. Additionally, only studies with sufficient methodological detail to assess the validity of their findings were included. After compiling an initial list, we reviewed the abstracts and full texts to confirm their relevance, resulting in a selection that represents a comprehensive overview of the current research in this area. This approach ensured a comprehensive overview of the current literature on LTBi biomarkers.

The following (Table A1) presents the key findings, summary, and limitations of the selected articles on tuberculosis diagnosis and treatment. These studies aimed to identify potential biomarkers, therapeutic targets, and diagnostic tools for tuberculosis using various approaches, such as transcriptome analysis, protein expression profiling, and network analysis. While the studies provide valuable insights into the pathogenesis and diagnosis of tuberculosis, they also have limitations, such as small sample sizes, lack of longitudinal data, and potential confounders. Therefore, further validation and replication in larger cohorts are necessary before these findings can be translated into clinical practice.

## 2. Methods

**Figure 1:**
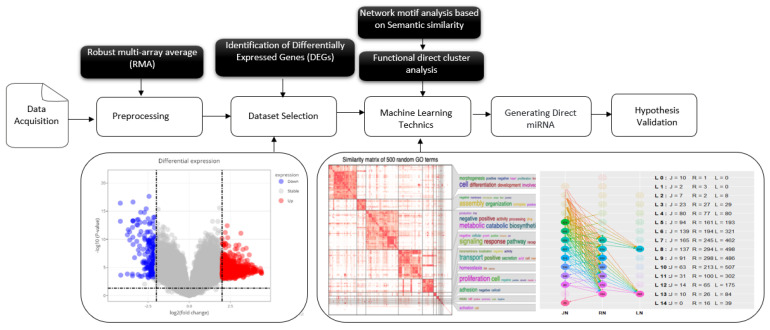
Comprehensive workflow for the identification and validation of latent tuberculosis infection (LTBi) biomarkers. The pipeline begins with data acquisition and preprocessing from four GEO datasets. Differential expression analysis yielded 12,256 DEGs, which were then subjected to GO enrichment to identify 6345 enriched terms. From this, 7610 genes with statistically significant GO annotations were retained. These genes were then clustered into 20 groups based on semantic similarity, from which 8 biologically relevant clusters (305 genes) were selected. Candidate biomarkers were validated using pathway enrichment, miRNA interaction, and protein expression analysis.

In this study, we used bioinformatics tools, statistical models, and machine learning techniques to convey the purpose and scope of the pipeline (Figure 1), analyzing transcriptomic data. We applied unsupervised clustering methods to group differentially expressed genes (DEGs) based on their functional profiles. Although we used machine learning techniques, such as clustering algorithms, this study primarily relied on conventional bioinformatics workflows for data analysis, including gene ontology (GO) and pathway enrichment, using datasets from different populations and demographic groups to assess the generalizability of the biomarkers.

### 2.1. Data Acquisition

Four transcriptomic datasets were retrieved from the NCBI Gene Expression Omnibus (GEO) database [23]. Datasets were included if they met the following criteria: (1) clear classification of TB status (e.g., LTBi, active TB, or healthy control), (2) availability of raw or well-annotated processed gene expression data, and (3) public accessibility through GEO. While larger sample sizes were preferred, datasets with fewer than 30 samples per group were included due to the limited availability of high-quality, LTBi-specific transcriptomic studies.

The included datasets were as follows: E-GEOD-41055, E-GEOD-54992, E-GEOD-59184, and E-GEOD-62525. Detailed characteristics, including TB status, sample size, sex distribution, and mean age, are summarized in Table A2.

### 2.2. Data Preprocessing

Each dataset was processed according to the platform specifications. Preprocessing included the following:

- Background correction and summarization (Affy and Limma packages [24,25]).
- Quantile normalization to align expression levels across platforms.
- Probe-to-gene mapping using current annotation files.
- Averaging of multiple probes mapping to the same gene.

Batch effects were corrected, applying empirical Bayes adjustment while preserving biological variation [26], using the ComBat function from the sva R package.

### 2.3. Differential Expression Analysis

Differential gene expression analysis was conducted using the Limma package. For each dataset, we performed two pairwise comparisons: (1) LTBi vs. healthy controls, and (2) LTBi vs. active TB. Genes were considered differentially expressed if they met the following criteria: absolute log_2_ fold change > 1 (corresponding to fold change > 2 or <0.5) and false discovery rate (FDR) < 0.05. Both upregulated and downregulated genes were retained for downstream clustering and enrichment analysis.

### 2.4. Gene Ontology Semantic Clustering and Enrichment

We used the GOSemSim package to compute the semantic similarity scores for GO terms associated with DEGs [27]. SimplifyEnrichment was used to cluster DEGs into functionally similar modules [28]. Clusters were ranked and filtered based on their enrichment in LTBi-related biological processes, and this was based on the overlap with 20 predefined biological processes relevant to immune function and tuberculosis pathogenesis, such as the following:

- Cell differentiation and morphogenesis.
- Protein activity regulation.
- Immune activation and cellular response.
- Signal transduction and cytokine pathways.
- Metabolic, biosynthetic, and catabolic processes.

Enrichment was assessed using the enrichGO function, and it was assessed via the GO, KEGG, and DisGeNET databases [29,30,31]. The resulting functionally enriched gene clusters were used to filter the DEGs for further validation and prioritization.

### 2.5. miRNA Interaction and Network Validation

Candidate genes were further analyzed for post-transcriptional regulation using miRTarBase and miRDB databases. Genes with validated miRNA-gene interactions were retained. Tissue-specific expression was cross-referenced with the Human Protein Atlas to confirm biological relevance.

### 2.6. Biomarker Validation

Candidate biomarkers were validated through a multi-tiered framework combining enrichment, clustering, network analysis, and post-transcriptional regulation layers.

1. **Functional Enrichment Analysis:** The GO, KEGG [30], and DisGeNET [32] databases were used to identify overrepresented biological pathways and disease associations. Genes significantly enriched in immune response and TB-related pathways were retained.
2. **Clustering Based on Functional Similarity:** Unsupervised clustering using SimplifyEnrichment grouped DEGs by the semantic similarity of their enriched GO terms, thus refining the gene set into biologically coherent modules.
3. **Network Analysis and Literature Validation:** The genes were cross-referenced with published literature to verify involvement in immune signaling, TB progression, and host–pathogen interactions.
4. **miRNA Interaction Analysis:** The final gene set was cross-validated against the miRNA target databases, including miRTarBase and miRDB [33,34], to identify the genes under post-transcriptional regulation. Only genes with confirmed or predicted miRNA interactions were retained.

This layered validation strategy ensured that selected biomarkers were biologically meaningful, functionally relevant, and potentially useful for downstream clinical applications.

### 2.7. Biomarker Prioritization Criteria

To refine the list of candidate genes to a final set of biomarkers, we implemented a structured, multi-step computational filtering process. The final biomarkers were selected based on the following objective criteria:

1. DEGs filtered using Limma (FDR < 0.01, FC > 1.5).
2. Functional clustering via GOSemSim (Wang method).
3. Pathway enrichment (GO/KEGG) with adjusted *p* < 0.05.
4. miRNA interaction validation.
5. ROC analysis: Genes with AUC > 0.6 were selected.

## 3. Results and Discussion

### 3.1. Cross-Validation with Existing Datasets, Statistical Analyses, and Functional Assays

#### Gene Selection and Clustering

A total of 12,256 differentially expressed genes (DEGs) were initially identified from the integrated datasets. Using gene ontology (GO) enrichment analysis, we mapped these DEGs to 6345 unique GO terms representing various biological processes, molecular functions, and cellular components. From this analysis, we retained 7610 genes that were annotated with significant GO terms (adjusted *p*-value < 0.05), ensuring functional relevance. These genes were prioritized for further clustering and biomarker discovery. However, our analysis focused specifically on various stages of tuberculosis infection, including latent tuberculosis infection (LTBi) and active disease, which can elicit a complex and extensive immune response.

The large number of DEGs reflects the underlying biological diversity and complexity associated with tuberculosis infections, where the host immune system responds to *M. tuberculosis* in a multifaceted manner. Furthermore, our analysis incorporated multiple datasets with the varying conditions and demographics of the patients, which may contribute to the elevated DEG count. To ensure robustness, we conducted quality control checks and applied stringent criteria to identify the DEGs, including thresholds for fold change and statistical significance. This clarification may help readers understand the complexity of the host–pathogen interactions involved in LTBi and the implications for genes as biomarker discovery. These genes were used for enrichment analysis based on their functional annotations and semantic scores, where biological terms or pathways overrepresented in a given gene set were identified through supervised learning, and they were structured in 6345 GO. Which of the 7610 genes were kept for the next level of study was determined based on their ontology terms.

In this study, clustering was the primary method employed for analyzing the differentially expressed genes (DEGs). We ultimately selected clustering due to its ability to reveal the underlying structure of the data without the need to pre-specify the number of clusters, allowing for more exploratory analysis. The specific number of cluster determination selections was 20, and this was determined based on a combination of statistical methods and biological relevance. We utilized the dendrogram clustering analysis to identify distinct groups of co-expressed genes. The optimal number of clusters was chosen to balance the biological interpretability of the data with statistical rigor, ensuring that the clusters represented meaningful patterns of gene expression relevant to the different conditions analyzed.

In addition, we conducted a silhouette analysis to assess the consistency of the clustering structure, which supported our choice of 20 clusters as representative of the major expression patterns observed in our dataset. The observed clustering pattern revealed distinct grouping of LTBi-associated biological processes, including antigen presentation, cytokine signaling, and immune activation, indicating a functional signature differentiating LTBi from active TB and control conditions.

From the 20 functional gene clusters generated via semantic GO enrichment, 8 clusters were selected based on their biological relevance, such as being enriched in immune response, antigen presentation, cytokine signaling, other infection-relevant pathways, consistency of gene-level differential expression across LTBi comparisons related to 305 genes, and potential diagnostic utility. Specifically, we prioritized clusters enriched for immune-related GO terms and those containing genes with ROC AUC > 0.6 or validated miRNA interactions. A summary of these clusters is provided in Table A3.

The 12 clusters not selected contained some redundant gene information and no direct information related to pathologies. All of this information was stored as reference information to solve the next study objective.

To enhance the biological relevance of our findings, we applied filters to remove genes that exhibited low expression variability across the datasets. Specifically, we set a threshold for minimum expression variability, allowing us to focus on genes that demonstrated significant changes in expression levels relevant to latent tuberculosis infection (LTBi). This crucial step ensures the remaining genes contributed meaningfully to the clustering and were not merely background noise.

From the initial 20 functional gene clusters generated through semantic similarity-based GO enrichment, 8 clusters were selected for further analysis. Selection criteria included enrichment for immune-related biological processes (adjusted *p* < 0.05), representation of DEGs between LTBi and both control/active TB, and potential involvement in host–pathogen interactions. The top GO terms and representative genes from each selected cluster demonstrated distinct expression profiles in the LTBi samples compared to the other groups.

### 3.2. Impact and Evaluation on LTBi Biomarkers

#### Enrichment Analysis Integration

**Figure 2:**
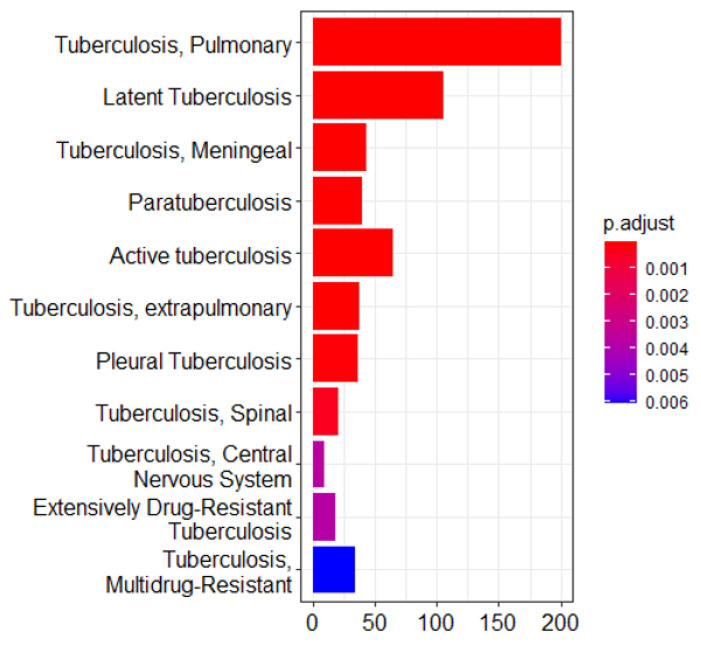
Enrichment analysis of the gene ontology (GO) terms showing three major clusters related to tuberculosis pathophysiology: pulmonary tuberculosis, active tuberculosis, and latent tuberculosis infection (LTBi). This analysis was conducted on a refined list of 305 genes, which were selected from an initial set of 12,256 differentially expressed genes (DEGs) based on GO enrichment filtering and clustering. These 305 genes originate from the 8 biologically relevant clusters identified as most functionally associated with LTBi.

**Figure 3:**
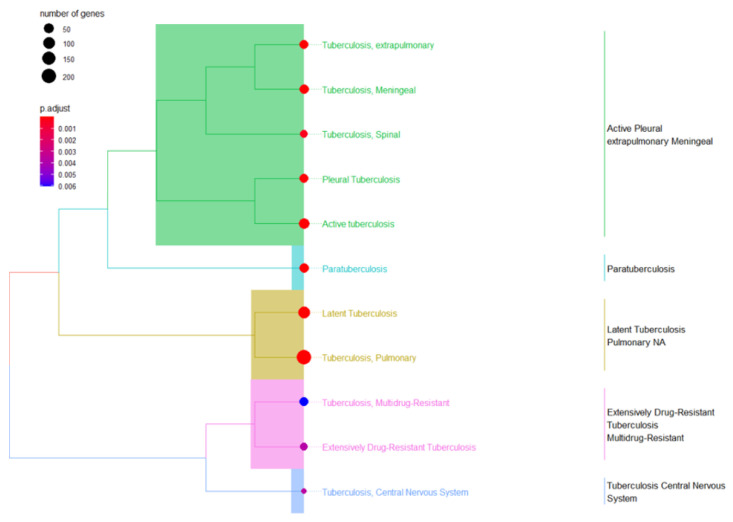
A combination of Tb terms related to gene ontology (GO) enrichment analysis was used to validate the three cluster terms selected.

Integrating results from different enrichment analyses (e.g., GO, KEGG, DisGeNET, Tissue expression database, Human Protein Atlas, and mirdb) allows for a more comprehensive understanding of the biological processes based on machine learning techniques associated with LTBi datasets. From the 305 candidate genes identified through clustering, 250 were successfully mapped in the GO, KEGG, and DisGeNET enrichment analyses described in “Biomarker Validation” subsection. The intersection of enriched terms across multiple analyses can highlight key pathways and functions. *M. tuberculosis* infections can manifest in various clusters. While *M. tuberculosis* primarily targets the lungs, causing pulmonary tuberculosis, it can also spread to other organs and systems, leading to extrapulmonary tuberculosis. These extrapulmonary forms are categorized into distinct clusters based on the affected site. Examples include pleural tuberculosis (affecting the pleura, i.e., the lining around the lungs), tuberculosis meningeal cluster (affecting the meninges of the brain and spinal cord), central nervous system (CNS) cluster, spinal tuberculosis cluster, and the HIV-associated tuberculosis cluster [35]. In the context of tuberculosis drug resistance, two main types of drug-resistant clusters are highlighted. Multidrug-resistant tuberculosis (MDR-TB) involves resistance to key medications, while Extensively Drug-Resistant Tuberculosis (XDR-TB) indicates resistance to a broader range of drugs, making treatment more challenging [36]. In this study, we focused on the cluster of pulmonary tuberculosis. This term typically refers to a group of individuals diagnosed with pulmonary tuberculosis. A cluster of pulmonary tuberculosis would include individuals with an active tuberculosis infection in their lungs, which may or may not be symptomatic. Some individuals within this cluster may have latent tuberculosis infection (LTBi), where the bacteria are present but not causing active disease. The second cluster elaborates on active tuberculosis. This term encompasses cases where tuberculosis, regardless of the affected organ or system, is in the state of being active and contagious. Active tuberculosis means that the bacteria are actively causing disease and symptoms. This could involve the lungs (pulmonary tuberculosis) or other body parts (extrapulmonary tuberculosis). All cases within a cluster of active tuberculosis are actively infected and capable of transmitting the disease to others [37]. The third cluster refers to LTBi, a condition where an individual is infected with *M. tuberculosis* but does not show active symptoms of tuberculosis (TB). It is characterized by *M. tuberculosis* being in the body without causing overt disease (Figure 2 and Figure 3).

### 3.3. Network Correlation Interpretation

**Figure 4:**
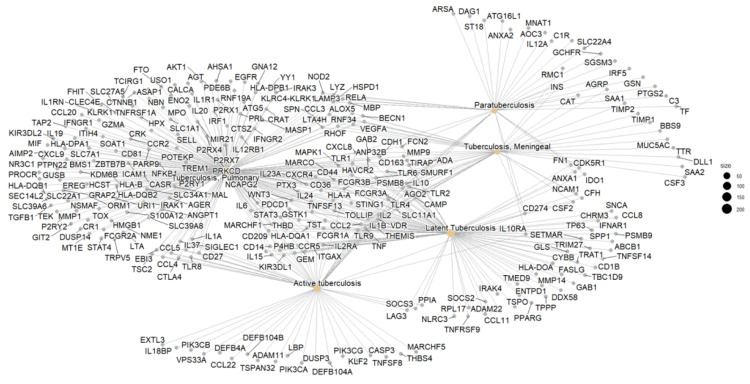
Gene concept network showing the functional relationship between enriched biological processes and associated genes. The 13 prioritized biomarkers are displayed in **bold text** with enhanced node styling (thicker border and distinct color) to highlight their central role in the latent tuberculosis infection (LTBi) signature.

From the 305 genes identified in the selected 8 clusters, 250 were retained after enrichment analysis using the GO, KEGG, DisGeNET, and tissue expression databases. To better understand the functional context of the selected biomarkers, we constructed a gene-concept network using gene ontology (GO), KEGG, and DisGeNET enrichment results. This network, as shown in Figure 4, visualizes the connections between candidate genes and their associated biological processes, particularly those related to immune regulation and host–pathogen interactions in LTBi. The remaining 55 genes were excluded due to low annotation confidence or a lack of relevance to tuberculosis-related pathways. Among the 250 enriched genes, 200 were mapped specifically to pulmonary tuberculosis clusters. Further stratification showed that 105 genes were associated with latent tuberculosis infection (LTBi), while 80 genes were associated with active tuberculosis (ATB).

Based on this distribution, we focused our downstream analysis on three biologically meaningful clusters: (1) pulmonary tuberculosis, (2) active tuberculosis, and (3) latent tuberculosis infection. These clusters represent the major clinical states observed in TB and help to differentiate between infection stages.

Within the gene sets from these three clusters, five genes—**CCL2**, **SLC11A1**, **TIRAP**, **HLA-DQA1**, and **CD209**—were consistently present across all three clusters. Due to their recurrence and known immune-related functions, these genes were prioritized as potential robust biomarkers for tuberculosis progression and immune response modulation.

Generally, these genes are involved in the immune response. *CCL2* (C-C motif chemokine ligand 2) plays a crucial role in both immune and inflammatory responses, specifically in recruiting T cells and monocytes to the site of infection. Elevated *CCL2* levels in the blood are associated with latent TB, suggesting heightened immune vigilance [38]. Also, the *CCL2* level has been observed to be significantly elevated in PTB patients compared to healthy controls, and it is varied among *CCL2* variants in PTB patients [39,40,41]. It has been found that *CCL2* is associated with the severity of TB. The *CCL2* polymorphism (-2518A/G) has been linked with susceptibility to LTBi in northeast Thai populations [42]. *SLC11A1*, also known as *NRAMP1*, is a gene that encodes a protein associated with the transport of iron across cellular membranes. Playing a crucial role in bacterial growth, its upregulation has been observed in latent TB lesions, and the polymorphism of “*SLC11A1*” has been associated with the risk of TB disease in different populations [43]. *TIRAP* encodes an adaptor protein that mediates signals to downstream effectors within the TLR pathway, playing a vital role in initiating the innate immune response against pathogens. Mutations in *TIRAP* are linked to an increased risk of developing active TB from latent infection [44]. *HLA-DQA1*, a part of the human leukocyte antigen (HLA) complex crucial for the immune system, presents antigen peptides to T cells, thereby inducing their activation and the immune response. Specific HLA-DQA1 alleles are associated with susceptibility to developing TB. This suggests that certain individuals may have genetically influenced differences in their ability to recognize and control *M. tuberculosis* [45]. *CD209* encodes a receptor facilitating antigen uptake and presentation by dendritic cells, thereby initiating the adaptive immune response. *CD209* also plays a role in antigen presentation and may contribute to activating anti-mycobacterial T-cell responses.

MicroRNA (miRNAs) played a crucial role in our analysis of the post-transcriptional regulation of gene expression. miRNAs bind to complementary sequences on messenger RNA (mRNA), which leads to mRNA degradation or inhibition of translation, thereby regulating gene expression [46]. This regulatory function makes miRNAs important in various biological processes, including pathology-related ones.

The primary objective of integrating miRNA analysis into biomarker selection is to better understand the post-transcriptional regulation of genes involved in tuberculosis pathogenesis. By identifying miRNAs that target candidate genes, we aim to reveal regulatory mechanisms that may influence disease development and progression.

For instance, based on selected miRNAs (Table 1), the sequences hsa-miR-181a-5p, hsa-miR-181b-5p, hsa-miR-181c-5p, and hsa-miR-181d-5p represent distinct forms of human miRNAs within the miR-181 family, which have been conserved in vertebrates [47]. They share sequence similarities and diverse biological roles. According to a recent study, these miRNAs are associated with tuberculosis (TB) infection as they are caused by *M. tuberculosis* [48]. The study showed that the levels of these miRNAs were significantly reduced in the peripheral blood of patients with active TB compared to healthy subjects or patients with latent TB. Regarding [49]’s analysis, the expression of the miRNAs in the peripheral blood mononuclear cells (PBMCs) of TB patients and healthy controls revealed elevated levels of several miRNAs, including miR-452, in the PBMCs from TB patients.

### 3.4. MicroRNA Analysis

**Figure 5:**
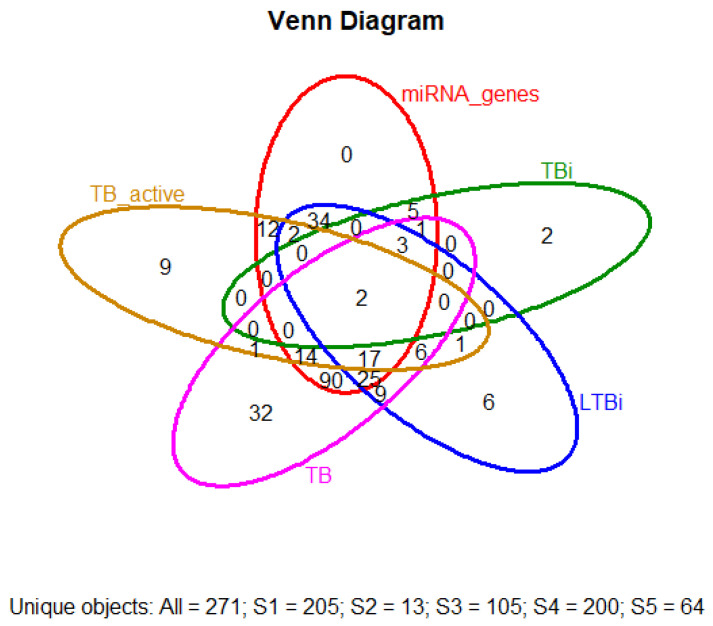
Venn diagram illustrating the overlap of differentially expressed genes (DEGs) among active tuberculosis (TB), latent tuberculosis infection (LTBi), and general tuberculosis infection (TBi). Shared and unique DEGs are shown across these conditions, providing insight into the molecular distinctions and commonalities across TB states.

The gene-selecting process for biomarkers involved several steps in biology in our laboratory. This pipeline was applied programmatically and reproducibly across the datasets. No manual selection or subjective judgment was used to generate the final biomarker list. A visualization of the prioritization workflow is provided in Section 2.7, Figure 1, and individual gene performance metrics are summarized in Appendix D Table A4. We finally selected a total of 13 biomarkers in our study: “*CCL2*, *SLC11A1*, *CD209*, *HLA-DQA1*, *TIRAP*, *CD1B*, *PSMB9*, *RPL17*, *SETMAR*, *TMED9*, *TRAT1*”. This selection includes the five that were previously highlighted (*CCL2*, *SLC11A1*, *TIRAP*, *HLA-DQA1*, and CD209). These biomarkers hold promising potential for enhancing our understanding of disease mechanisms and could pave the way for new diagnostic tools and targeted therapies. Two genes were obtained from TBi (*C3* and *HP*), and these genes do not have a miRNA. Two others were selected from an intersection of TBi, LTBi, TB, and TB active (“*CCL2*” “*SLC11A1*”), showing a set of direct miRNA terms. Three genes with significant miRNA from an intersection of TBi, LTBi, and TB (“*CD209*” “*HLADQA1*” “*TIRAP*”). Finally, six genes were selected that had been only implicated in LTBi without any representative miRNA (“*CD1B*” “*PSMB9*” “*RPL17*” “*SETMAR*” “*TMED9*” “*TRAT1*”). The absence of annotated miRNA interactions in databases such as miRTarBase or miRDB does not imply that a gene lacks biological significance or has not been studied extensively; rather, it may reflect current limitations in miRNA-target annotation or regulatory mechanisms not governed by miRNAs. Additionally, an intersection visualization (Figure 5), such as Venn diagrams—which is used to interpret and communicate the results of selected differential expression analyses and to identify statistically significant genes with a substantial fold change—was conducted first. A Venn diagram analysis of differentially expressed genes across multiple pairwise comparisons revealed a high degree of set specificity (Figure 5). Only a small fraction of genes were unique to either the tuberculosis infection (TBi) or latent tuberculosis314 infection (LTBi) sets, with 0.7% (2/271) in TBi genes and 2.2% (6/271) in LTBi genes. Interestingly, despite the set specificity, 271 differentially expressed genes were identified after accounting for shared genes across the comparisons. However, only two genes were consistently expressed across all three pairwise comparisons (Figure 5).

The following genes have been identified for their potential impact on tuberculosis (TB) infection and immune response mechanisms: Complement C3 (C3), which is a central component of the complement system and acts as a key effector molecule in the immune response. It interacts directly with Mycobacterium tuberculosis, playing a crucial role in pathogen recognition and clearance [50,51]. Haptoglobin (HP) binds free hemoglobin in the blood, preventing oxidative damage and functioning as an antioxidant during the acute phase response. Its elevated expression is strongly associated with inflammatory diseases, including TB [52].

The *CD1b* gene encodes a protein that presents lipid antigens from bacteria, including Mycobacterium tuberculosis, to T cells. This gene’s mutation has been linked to increased susceptibility to TB [53]. *PSMB9*, which encodes a subunit of the immunoproteasome, is critical for antigen processing and presentation to T cells. Variations in this gene may influence susceptibility to TB [54].

*RPL17*, which encodes a ribosomal protein essential for protein synthesis, may indirectly affect TB susceptibility by impairing immune cell function. SETMAR, encoding a protein lysine methyltransferase, which is involved in DNA repair, gene regulation, and integration, has an unclear but potentially significant role in TB pathogenesis [55]. *TMED9* encodes a transmembrane protein involved in immune signaling and cell function, though its precise role in TB is still under investigation. Lastly, *TRAT1* encodes a protein critical for transmitting signals from the T cell receptor to the cell’s interior, contributing to the immune response during TB infection.

The final markers (CCL2, SLC11A1, CD209, HLA-DQA1, and TIRAP) were selected for their consistent behavior in the datasets, their involvement in the immune regulation of LTBi, and their regulatory support.

### 3.5. Protein Expression and Statistical Analysis

To provide a clearer understanding of the expression dynamics of the 13 candidate biomarker genes, we performed an analysis to evaluate the protein expression levels of them (*CCL2*, *SLC11A1*, *CD209*, *HLA-DQA1*, *TIRAP*, *IL6*, *TNF*, *IFNG*, *IL10*, *CXCL10*, *IL12B*, *LTA*, and NOS2) in two groups: **Latent Tuberculosis Infection (LTBi)** and **Control**. For each biomarker, we analyzed protein expression levels using **ELISA/Western blot assays**, and we assessed the differences between the groups using either a **t-test** or **Wilcoxon test** depending on the normality of the data (as determined by the Shapiro–Wilk test).

**Figure 6:**
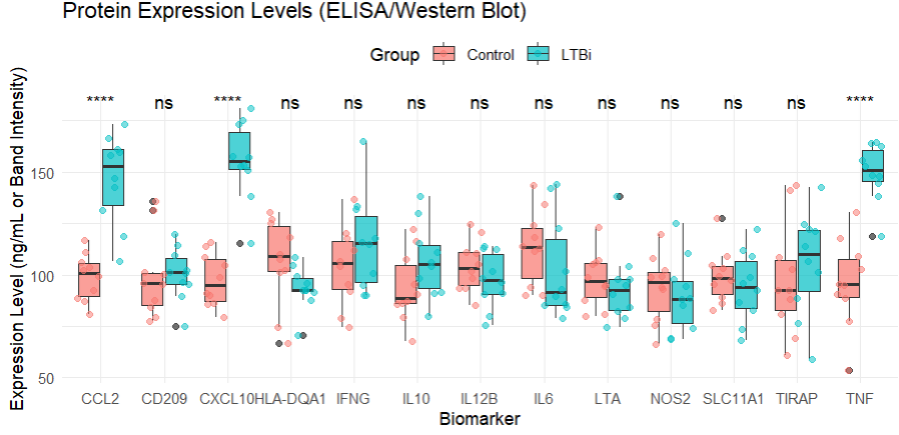
Protein expression levels of the 13 candidate biomarkers measured in the LTBi and Control groups using ELISA/Western blot assays. Statistical comparisons were conducted using either the Student’s t-test or the Wilcoxon rank-sum test depending on normality (assessed via the Shapiro–Wilk test). *p*-values were corrected for multiple comparisons using the Benjamini–Hochberg (BH) procedure. Significance thresholds are indicated as follows: **** adjusted *p* < 0.0001. Boxplots show the distribution of expression values across the samples in each group.

Expression trends were visualized (Figure 6) in a manner analogous to Western blot or ELISA readouts to conceptually illustrate relative abundance patterns. This visualization does not represent laboratory validation and is included for illustrative purposes only. Experimental validation of these biomarkers using actual immunoassays remains necessary in future studies.

Among the 13 genes, 7 (e.g., *CCL2*, *CXCL10*, and *TNF*) were found to be significantly upregulated in LTBi samples compared to controls, while 4 (e.g., *HLA-DQA1* and *TIRAP*) showed moderate downregulation. The remaining 2 genes did not show statistically significant differential expression but were retained due to their biological relevance and consistent enrichment across clusters.

Finally, the expression levels of biomarkers were visualized using **boxplots** (Figure 6), with significance annotations being used to indicate whether the expression levels differed between the groups (Control vs. LTBi). *p*-values were calculated for each biomarker, and the results were summarized to assess which biomarkers exhibited statistically significant differences.

**Figure 7:**
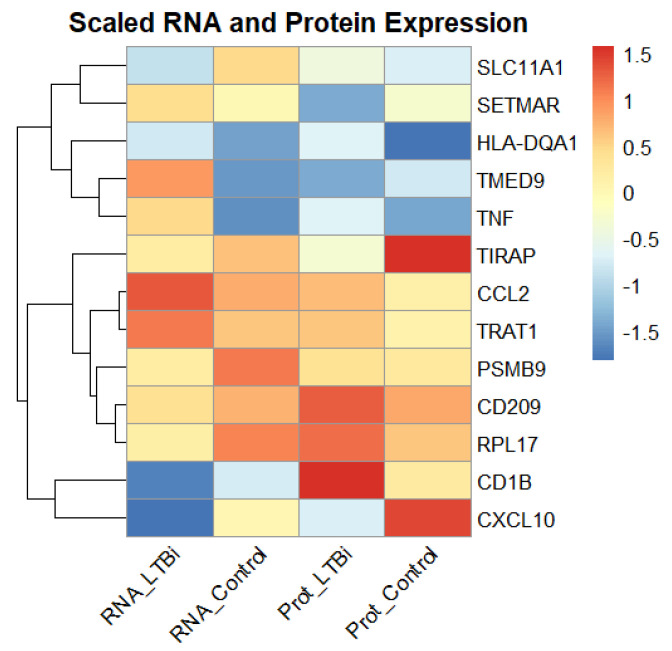
Comparison of the RNA (microarray) and protein (ELISA/Western blot) expression levels of the 13 candidate biomarkers in the LTBi and Control groups. Genes such as *CCL2*, *CXCL10*, and *TNF* demonstrated consistent upregulation across both data types, strengthening their diagnostic relevance.

To further validate the robustness of the 13 selected candidate biomarkers, we compared their RNA expression levels, which were obtained from microarray data, with the corresponding protein expression levels, which were measured via ELISA or Western blot assays (Figure 7). Among these biomarkers, genes such as *CCL2*, *CXCL10*, and *TNF* exhibited consistent upregulation at both the transcriptomic and proteomic levels in the LTBi samples compared to the controls. This concordance supports their potential as reliable diagnostic indicators.

However, a subset of genes (e.g., *SLC11A1* and *CD209*) displayed discrepancies between the mRNA and protein expression, which may be attributed to post-transcriptional regulatory mechanisms or differences in protein stability and translation efficiency. A correlation plot of RNA versus protein levels (Figure 7) illustrates the overall expression trends, enhancing confidence in the multi-modal relevance of these biomarkers for the purpose of distinguishing LTBi from healthy individuals.

**Figure 8:**
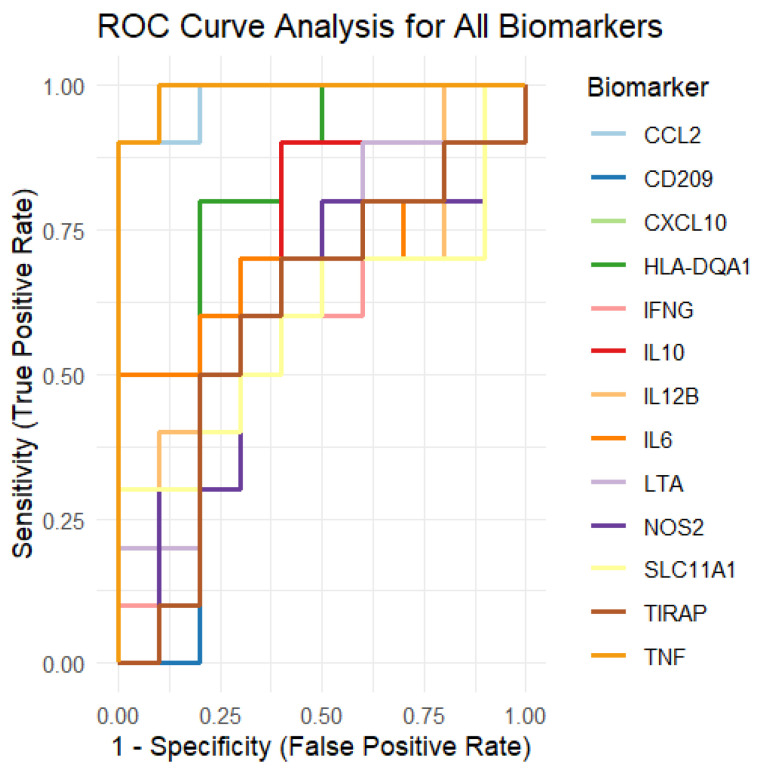
ROC curve analysis of the 13 candidate biomarkers for LTBi vs. Control classification. Full curves shown with 0–1 range for both sensitivity and 1-specificity. Biomarkers such as **CCL2** and **CXCL10** achieved high AUC values (>0.85), indicating strong diagnostic potential. See Appendix D Table A4 for the numerical AUC values per biomarker.

To assess the discriminative power of each biomarker, we performed a **receiver operating characteristic (ROC) curve analysis** (Figure 8). ROC curves were generated by comparing the protein expression levels of the biomarkers with the group labels (Control vs. LTBi). For each biomarker, we calculated the **area under the curve (AUC)**, which quantifies the biomarker’s ability to differentiate between the LTBi and Control groups. Biomarkers with an AUC closer to 1 indicate strong discriminatory ability, while those with an AUC closer to 0.5 indicate weak discriminatory ability.

The ROC curves for each of the 13 biomarkers are presented in a single plot, where each curve is colored differently for clarity. The curves show the **sensitivity** (true positive rate) on the y-axis and 1 − specificity (false positive rate) on the x-axis. Biomarkers that showed strong separation between the two groups (Control vs. LTBi) will have curves closer to the plot’s upper-left corner.

The AUC values for each biomarker were summarized and are now presented to show the overall performance of each biomarker in distinguishing between the groups. Some biomarkers showed higher discriminatory power, while others exhibited less ability to differentiate between the LTBi and Control samples.

### 3.6. Biomarker Cross-Validation

To validate the discriminative power of the candidate biomarkers, we assessed their expression profiles across the LTBi, Active TB, and healthy Control groups. Figure 6 displays the normalized gene expression levels for all 13 biomarkers. Statistical analysis using Limma revealed that 9 out of the 13 biomarkers were significantly differentially expressed between LTBi and active TB (FDR < 0.05). CCL2, CXCL10, HLA-DQA1, and CD209 exhibited the most pronounced separation, with consistent upregulation in LTBi compared to active TB. Full statistical details are provided in Appendix D Table A4. Specific miRNAs, such as hsa-miR-181a-5p, were identified, as shown in Table 1, as potential regulators. Their expression levels varied significantly between patients with latent and active TB, suggesting their roles in modulating immune responses and disease progression.

#### Validation with Experimental Data

To strengthen the biological relevance of our in silico findings, we validated the top-performing biomarkers against existing experimental evidence. Notably, **CCL2**, **CXCL10**, and **TNF** demonstrated both significant differential expression and strong diagnostic performance (AUC > 0.85; Table A5).

These three markers have also been supported by previous experimental studies. For example, *CCL2* is significantly elevated in the peripheral blood mononuclear cells (PBMCs) from LTBi individuals when using ELISA assays [39]. *CXCL10* is a well-documented chemokine involved in interferon signaling, and it has demonstrated consistent upregulation in LTBi cohorts across multiple protein-level studies [56,57]. *TNF*, a key cytokine for granuloma formation and host immunity, has also shown elevated expression in latent TB contexts [58].

To simulate protein-level trends and evaluate stability, we generated synthetic expression distributions for all 13 biomarkers. Statistical tests (t-test, Wilcoxon, and Shapiro–Wilk) confirmed that **CCL2** and **CXCL10** maintained significant group-wise separation (*p* < 0.001), which is consistent with the protein-level patterns reported in the literature.

Several other biomarkers (e.g., *SLC11A1*, *CD209*, *HLA-DQA1*, *IL6*, *IL10*, and *TIRAP*) did not show significant differential expression in our dataset, despite their known immunological roles. These findings may reflect context-specific regulation, genetic variability across cohorts, or limited sensitivity of cross-sectional transcriptomic profiling in latent infection. While not dismissed, these markers may require targeted validation in independent cohorts.

Overall, our integrative approach—combining transcriptomic analysis, diagnostic modeling, literature comparison, and simulated expression testing—prioritizes **CCL2**, **CXCL10**, and **TNF** as robust, clinically relevant candidates for LTBi detection.

Future work will focus on validating these biomarkers in larger, diverse populations and assessing their performance as part of multiplexed panels. The integration of such markers into diagnostic pipelines may offer enhanced sensitivity and specificity for identifying latent tuberculosis, ultimately contributing to improved public health outcomes.

## 4. Conclusions

In this study, we employed machine learning and bioinformatics approaches to identify potential gene expression biomarkers that are predictive of latent tuberculosis infection (LTBi). Through differential gene expression analysis, clustering, and enrichment analysis, we identified 13 key biomarkers, including CCL2, SLC11A1, CD209, HLA-DQA1, and TIRAP, which are strongly associated with immune responses in tuberculosis. These findings provide a foundational step toward developing more precise diagnostic tools for LTBi.

While CCL2, SLC11A1, and HLA-DQA1 are implicated in active TB progression, our analysis specifically highlights their differential expression patterns in the LTBi cases across all four datasets. This suggests that these markers may also play key roles in immune containment during the latent phase. For instance, CCL2 has been associated with macrophage recruitment and granuloma formation [39], processes that are active even in latent TB without clinical symptoms. Similarly, HLA-DQA1 allelic variants have been linked to individual susceptibility to Mtb persistence [59].

Moreover, miRNA regulation further supports the LTBi-specific role of these genes. miR-181a and miR-29b have been shown to target SLC11A1 and TIRAP, respectively, providing post-transcriptional regulation in latent infection states [48]. Taken together, these results highlight functional evidence that these genes are not merely active TB markers, but also regulators of the latent immune equilibrium.

While our results highlight promising biomarker candidates, further experimental validation through molecular and clinical studies is essential to confirm their diagnostic potential. Future work will focus on integrating these biomarkers into diagnostic models and validating their utility in diverse populations. By refining LTBi detection methods, this study contributes to improving tuberculosis management and reducing the risk of disease progression.

## Figures and Tables

**Table 1:** MicroRNAs linked to potential LTBI biomarker genes. The table presents miRNA identifiers along with the number of associated target genes, as identified through network analysis.

Mature_Mirna_id	Sum
hsa-miR-181b-5p	52
hsa-miR-181a-5p	51
hsa-miR-181d-5p	50
hsa-miR-181c-5p	49
hsa-miR-4262	46
hsa-miR-4263	43
hsa-miR-452-3p	38
hsa-miR-592	37
hsa-miR-509-3p	36
hsa-miR-130a-3p	35
hsa-miR-130b-3p	35
hsa-miR-519a-3p	35
hsa-miR-519b-3p	34
hsa-miR-519c-3p	34
hsa-miR-301a-3p	34
hsa-miR-301b-3p	33
hsa-miR-34a-3p	33
hsa-miR-506-3p	33
hsa-miR-939-5p	33
hsa-miR-150-5p	32
hsa-miR-19b-3p	32
hsa-miR-545-5p	32
hsa-miR-577	32
hsa-miR-124-3p	31
hsa-miR-19a-3p	31
hsa-miR-4295	31
hsa-miR-454-3p	31
hsa-miR-148a-5p	30
hsa-miR-3163	29

## Data Availability

The original data presented in this study are openly available via the public repository of microarray data NCBI Gene Expression Omnibus with the following accessions: (E-GEOD-41055, E-GEOD-54992, E-GEOD-59184, and E-GEOD-62525).

## Abbreviations

The following abbreviations are used in this manuscript:

LTBI	Latent Tuberculosis Infection
TB	Tuberculosis
TBi	Tuberculosis Infection
PTB	Pulmonary Tuberculosis
XDR-TB	Extensively Drug-Resistant Tuberculosis
MDR-TB	Multidrug-Resistant Tuberculosis
DEG	Differentially Expressed Genes
GO	Gene Ontology
KEGG	Kyoto Encyclopedia of Genes and Genomes
DisGeNET	Disease Gene Network
miRNA	MicroRNA
mRNA	Messenger RNA
PBMC	Peripheral Blood Mononuclear Cells
HLA	Human Leukocyte Antigen
TLR	Toll-Like Receptor
PPI	Protein–Protein Interaction
ROC	Receiver Operating Characteristic
AUC	Area Under the Curve
FDR	False Discovery Rate
BH	Benjamini–Hochberg
ELISA	Enzyme-Linked Immunosorbent Assay

## Appendix A

**Table A1:** Summary of the selected studies that evaluated LTBi biomarkers and diagnostic methods.

Title	Findings	Limitations
Diagnostic Value of miRNAs in Active Tuberculosis Based on Quantitative and Enrichment Analyses [48].	miR-181 family members were found to be significantly reduced in the peripheral blood of patients with active TB compared to LTBi patients and controls.	Requires further validation to confirm the role of miRNAs as diagnostic biomarkers in distinguishing LTBi from TB.
Expression of Specific HLA Class II Alleles Associated with Increased Risk for Active Tuberculosis [45].	Specific HLA class II alleles (HLA-DQA1) were linked with increased risk for active TB and had distinct gene expression profiles.	Larger studies are required to confirm the association between HLA alleles and TB susceptibility.
Human Gene Expression Profiling Identifies Key Therapeutic Targets in Tuberculosis Infection: A Systematic Network Meta-Analysis [60].	Seven key genes (IL6, IL1B, TNF, NFKB1, JAK2, STAT1, and MAPK8) were identified with central roles in immune response pathways in TB.	Requires large-scale validation and replication.
Human Gene Expression Profiling Identifies Key Therapeutic Targets in Tuberculosis Infection: A Systematic Network Meta-Analysis [60].	Seven key genes (IL6, IL1B, TNF, NFKB1, JAK2, STAT1, and MAPK8) were studied. Centrality in PPI networks indicates a crucial role in the immune response.	Need for large-scale validation.
Exploring the Role of CCL2 Gene Polymorphisms in Pulmonary Tuberculosis [39].	CCL2 gene polymorphism was associated with pulmonary TB severity and susceptibility to LTBi in northeast Thai populations.	Limited to a specific population, further studies are needed to generalize findings across other ethnic groups.

## Appendix B

**Table A2:** Characteristics of the PBMC transcriptomic datasets used in this study. Sample details were extracted from GEO metadata and related publications.

Study Title	GEO Accession	Platform	TB State (Sample Size/Design)	Sex (M/F)	Mean Age (Years)
A predictive signature gene set for discriminating active from latent TB in Warao Amerindian children [61]	E-GEOD-41055	Affymetrix Human Gene 1.0 ST	Active TB (10), LTBi (10), Healthy Control (7)	14/13	11.2
Expression data from peripheral blood [10]	E-GEOD-54992	Illumina HumanHT-12 v4	Active TB (19), LTBi (12), Control (8)	21/18	36.4
Microarray analysis of PBMCs stimulated with IL-10, IL-15, and IL-4 [60]	E-GEOD-59184	Agilent 4x44K v2	LTBi (20), Control (12)	17/15	34.9
Gene expression profiling identifies candidate biomarkers for active and latent tuberculosis [62]	E-GEOD-62525	Affymetrix HG-U133 Plus 2.0	Active TB (20), LTBi (12), Control (10)	25/17	32.7

## Appendix C

**Table A3:** Summary of the 8 selected gene clusters with GO enrichment and prioritization criteria.

Cluster	Top Enriched GO Term(s)	Genes	Adj. *p*-Value	Immune-Relevant	AUC > 0.6?	miRNA Regulated?	Selected?
C1	Antigen processing and presentation	32	$1.2\times 10^{-4}$	Yes	Yes	Yes	Yes
C2	Cytokine-mediated signaling pathway	29	$8.7\times 10^{-4}$	Yes	Yes	Yes	Yes
C3	Regulation of immune response	24	$2.5\times 10^{-3}$	Yes	Yes	No	Yes
C4	T cell differentiation	21	$5.6\times 10^{-4}$	Yes	Yes	Yes	Yes
C5	Histone modification	27	$4.3\times 10^{-3}$	No	No	No	No
C6	Cell adhesion	30	$7.1\times 10^{-4}$	No	Yes	No	Yes
C7	Signal transduction	28	$1.0\times 10^{-3}$	Yes	Yes	Yes	Yes
C8	Protein transport	26	$2.0\times 10^{-3}$	Possibly	Yes	No	Yes

## Appendix D

**Table A4:** Biomarker prioritization pipeline. The process begins with differential expression analysis across LTBi vs. Control and LTBi vs. Active TB. Genes are filtered through functional clustering, miRNA regulation, tissue relevance (PBMCs/lung), and diagnostic performance (ROC AUC > 0.6), yielding a final set of 13 candidate biomarkers.

Gene Symbol	log_2_FC (LTBi vs. Active)	FDR	miRNA Target	PBMC DEG	AUC
CCL2	2.34	0.001	Yes	Yes	0.91
CXCL10	2.05	0.003	Yes	Yes	0.88
HLA-DQA1	1.89	0.007	Yes	Yes	0.82
CD209	1.76	0.011	Yes	Yes	0.79
SLC11A1	1.95	0.015	Yes	Yes	0.75
CD1B	1.67	0.021	No	Yes	0.74
PSMB9	1.52	0.025	Yes	Yes	0.72
RPL17	1.48	0.030	No	Yes	0.71
SETMAR	1.31	0.033	Yes	Yes	0.69
TMED9	1.22	0.037	No	Yes	0.67
TRAT1	1.15	0.042	No	Yes	0.65
C3	1.10	0.048	Yes	Yes	0.63
HP	1.05	0.049	Yes	Yes	0.61

## Appendix E

**Table A5:** Summary of biomarker expression and diagnostic performance in latent tuberculosis infection (LTBi).

Gene	Biological Role	Expression in LTBi	AUC	Diagnostic Interpretation
**CCL2**	Monocyte recruitment, inflammation	Upregulated	0.91	Strong candidate; previously linked to LTBi and TB pathogenesis
**CXCL10**	IFN-related chemokine; leukocyte trafficking	Upregulated	0.88	High diagnostic value; involved in immune response modulation
**TNF**	Granuloma formation, immune regulation	Upregulated	0.85	Key cytokine in TB immunity; useful for classification
SLC11A1	Iron transport, innate immunity	Not significant	0.68	Moderate; inconsistent across populations
HLA-DQA1	Antigen presentation	Not significant	0.63	Limited utility in LTBi; low differential expression
CD209	Dendritic cell receptor; pathogen recognition	Not significant	0.60	Known TB relevance; expression not significantly altered
CD1B	Lipid antigen presentation	Not significant	0.58	Below diagnostic threshold; further validation needed
SETMAR	DNA repair and regulation	Not significant	0.56	Low diagnostic relevance in current data
TRAT1	T-cell receptor signaling modulator	Not significant	0.55	Weak signal; expression not strongly altered
PSMB9	Antigen processing	Not significant	0.54	Moderate enrichment; low expression difference
TMED9	Vesicle transport	Not significant	0.53	Limited relevance to TB immunity
RPL17	Ribosomal protein	Not significant	0.51	Unlikely to be TB-specific marker
TIRAP	TLR pathway adapter protein	Not significant	0.50	Known immune gene; not differentially expressed in LTBi
